# Analysis of *Babesia bovis* infection-induced gene expression changes in larvae from the cattle tick, *Rhipicephalus (Boophilus) microplus*

**DOI:** 10.1186/1756-3305-5-162

**Published:** 2012-08-07

**Authors:** Andrew M Heekin, Felix D Guerrero, Kylie G Bendele, Leo Saldivar, Glen A Scoles, Cedric Gondro, Vishvanath Nene, Appolinaire Djikeng, Kelly A Brayton

**Affiliations:** 1 USDA-ARS, Knipling Bushland US Livestock Insect Research Laboratory, 2700 Fredericksburg Rd, Kerrville, TX, 78028, USA; 2 Department of Mathematics, University of Texas at El Paso, El Paso, TX, 79968, USA; 3 USDA-ARS Animal Disease Research Unit, Pullman, WA, 99164, USA; 4 The Institute for Genetics and Bioinformatics, University of New England, Armidale, NSW, 2351, Australia; 5 International Livestock Research Institute (ILRI) and Biosciences eastern and central Africa (BecA) Hub, PO Box 30709, Nairobi, Kenya; 6 Program in Vector-Borne Diseases, Department of Veterinary Microbiology and Pathology, Washington State University, Pullman, WA, 99164, USA

**Keywords:** Cattle tick, Rhipicephalus (Boophilus) microplus, Babesia bovis, Larva, Transcriptome, Serine proteinase inhibitor

## Abstract

**Background:**

Cattle babesiosis is a tick-borne disease of cattle that has severe economic impact on cattle producers throughout the world’s tropical and subtropical countries. The most severe form of the disease is caused by the apicomplexan, *Babesia bovis,* and transmitted to cattle through the bite of infected cattle ticks of the genus *Rhipicephalus*, with the most prevalent species being *Rhipicephalus (Boophilus) microplus*. We studied the reaction of the *R. microplus* larval transcriptome in response to infection by *B. bovis*.

**Methods:**

Total RNA was isolated for both uninfected and Babesia bovis-infected larval samples. Subtracted libraries were prepared by subtracting the B. bovis-infected material with the uninfected material, thus enriching for expressed genes in the B. bovis-infected sample. Expressed sequence tags from the subtracted library were generated, assembled, and sequenced. To complement the subtracted library method, differential transcript expression between samples was also measured using custom high-density microarrays. The microarray probes were fabricated using oligonucleotides derived from the Bmi Gene Index database (Version 2). Array results were verified for three target genes by real-time PCR.

**Results:**

Ticks were allowed to feed on a *B. bovis*-infected splenectomized calf and on an uninfected control calf. RNA was purified in duplicate from whole larvae and subtracted cDNA libraries were synthesized from *Babesia*-infected larval RNA, subtracting with the corresponding uninfected larval RNA. One thousand ESTs were sequenced from the larval library and the transcripts were annotated. We used a *R. microplus* microarray designed from a *R. microplus* gene index, BmiGI Version 2, to look for changes in gene expression that were associated with infection of *R. microplus* larvae. We found 24 transcripts were expressed at a statistically significant higher level in ticks feeding upon a *B. bovis*-infected calf contrasted to ticks feeding on an uninfected calf. Six transcripts were expressed at a statistically significant lower level in ticks feeding upon a *B. bovis*-infected calf contrasted to ticks feeding on an uninfected calf.

**Conclusion:**

Our experimental approaches yielded specific differential gene expression associated with the infection of *R. microplus* by *B. bovis*. Overall, an unexpectedly low number of transcripts were found to be differentially expressed in response to *B. bovis* infection. Although the BmiGI Version 2 gene index (http://compbio.dfci.harvard.edu/tgi/cgi-bin/tgi/gimain.pl?gudb=b_microplus) was a useful database to help assign putative function to some transcripts, a majority of the differentially expressed transcripts did not have annotation that was useful for assignment of function and specialized bioinformatic approaches were necessary to increase the information from these transcriptome experiments.

## Background

The cattle tick, *Rhipicephalus (Boophilus) microplus*, is a one-host tick parasitizing cattle in most of the world’s tropical and subtropical countries. This tick has a huge impact on cattle producers, large and small, with losses due to *R. microplus* infestations in Brazil alone estimated to be over $2 billion annually [1]. Perhaps the major impact is due to losses attributable to pathogens and their associated diseases transmitted by the tick’s bite. *R. microplus* is known to frequently harbor *Anaplasma marginale*, the causative agent of anaplasmosis, and *Babesia bovis* and *Babesia bigemina*, the apicomplexan agents that cause cattle babesiosis. The cattle-*R. microplusBabesia* complex has been described as the most important agricultural host-arthropod-pathogen complex globally [2]. *B. bovis* is generally responsible for the more serious cases of bovine babesiosis, with infection of naive hosts often causing pulmonary edema, central nervous system problems, and death. The severity of this disease in Australia is such that an anti-*B. bovis* vaccine is in widespread use across northern Australia in response to seasonal outbreaks in the major cattle producing areas [3].

When *R. microplus* ingests blood from a *B. bovis*-infected animal, the ingested merozoites undergo developmental changes in the tick midgut until the zygote stage of the apicomplexan enters the digestive cells of the tick’s gut where further multiplication and development to the kinete stage occurs. This kinete stage eventually enters the hemolymph and spreads to the rest of the tick’s tissues [3]. We are interested in transcriptional changes that accompany the various stages of infection by *B. bovis* as it interacts with its tick host. As our biological system, we allowed *R. microplus* to feed upon a splenectomized calf suffering from bovine babesiosis due to infection with *B. bovis*.We compared gene expression in larvae that hatched from eggs oviposited by adult female ticks that had fed on this infected calf with gene expression in larvae that hatched from eggs oviposited by adult female ticks that had fed on an uninfected calf. Our approaches included sequencing subtracted libraries synthesized from infected larvae, microarray analysis, and quantitative real-time polymerase chain reaction (qRT-PCR) to identify and annotate transcripts associated with *B. bovis* infection of *R. microplus* larvae.

## Methods

### Ticks

*R. microplus* larvae were from the f16 generation of the *B. bovis*-free La Minita strain. The La Minita strain was started from engorged female ticks collected from an outbreak in Starr County, Texas in 1996 and propagated at the USDA Cattle Fever Tick Research Laboratory at Moore Field, Texas before being transferred to the USDA-ARS Animal Disease Research Unit (ADRU) in Pullman, WA. Two splenectomized Holstein calves, 5-6 months of age, were used in these studies. One of the calves was infected by inoculating with frozen blood stabilate of the Texas Strain (2^nd^ passage), of *B. bovis.* The infection status of this calf was verified by blood testing and subsequently used to provide *B. bovis* infected tick larvae for this study. The second calf was maintained infection-free and was used to provide uninfected tick larvae for this study. All animal use was conducted at ADRU facilities at the University of Idaho Holm Research Center (Moscow, ID) while following protocols approved by the University of Idaho Institutional Animal Care and Use Committee.

*B. bovis*-infected larvae were obtained by placing uninfected larvae that hatched from 1.0 g of eggs from the *B. bovis*-free La Minita colony ticks into feeding patches glued to the shaved back and sides of the *B. bovis*-infected calf (designated as day 1). After feeding, all larvae were removed before they molted to the nymphal stage and frozen at -80^o^C immediately.

Uninfected larvae were obtained by placing uninfected larvae that hatched from 1.0 g of eggs from the *B. bovis*-free La Minita colony ticks into feeding patches glued to the shaved back and sides of the infection-free calf (designated as day 1). After feeding, all larvae were removed before they molted to the nymphal stage and frozen at -80^o^C immediately.

### RNA protocols

Separately, for both the uninfected and *Babesia bovis*-infected larval samples, total RNA was isolated from a pool of several thousand tick larvae using the FastPrep-24 Tissue and Cell Homogenizer and Lysing Matrix D (Qbiogene, Irvine, CA, USA) as described in Saldivar *et al.* (2008) [4]. Total RNA was treated with Turbo DNAse per Turbo DNA-free kit protocols (Ambion Inc.). RNA integrity was verified by formaldehyde gel electrophoresis and staining in GelStar Nucleic Acid Gel Stain (Lonza, Rockland, ME, USA). Twenty μg of DNA-free total RNA was sent to NimbleGen Systems Inc. (Madison, WI, USA) for use in microarray hybridization. Express Genomics Inc. (Frederick, MD, USA) was used to produce subtracted libraries from 0.15 mg each of *B. bovis*-infected and uninfected larval total RNA. The subtracted libraries, produced by subtracting the *B. bovis*-infected material with the uninfected material, were enriched for expressed genes in the *B. bovis*-infected sample.

### Sequencing and bioinformatics

Library sequencing was performed at the J. Craig Venter Institute (Rockville, MD). Bacterial colonies were picked for template preparation using colony-picking robots (Genetix, Boston, MA), inoculated into 384 well plates containing liquid medium and grown overnight. A robotic workstation was used to prepare sequencing grade plasmid DNA using an alkaline lysis method modified for high throughput processing [5]. Beckman Multimek 96 or Biomek FX automated pipetting robot work stations (Beckman Coulter, Fullerton, CA) were used to combine pre-aliquoted templates and reaction mixes consisting of deoxy- and fluorescently labeled dideoxynucleotides, *Taq* DNA polymerase, sequencing primers, and sequencing reaction buffer. Linear amplification steps were performed on MJ Research Tetrads PTC-225 (MJ Research, Inc., Watertown, MA) and sequencing reaction products purified by ethanol precipitation and resolved on ABI 3730xl sequencing machines (Applied Biosystems, Foster City, CA).

The larval subtracted library expressed sequence tags (ESTs) were assembled into 469 contigs with Paracel Transcript Assembler (2002) with the default assembly parameters (http://www.paracel.com/), and submitted to GenBank TSA with accession numbers JT844344-JT844812. The remaining 306 singleton reads, representing paired-end sequences that did not assemble, were submitted to GenBank dbEST with the accession numbers of FG579553-FG579858. All ESTs were screened for vector contamination with the SeqClean vector trimming utility downloaded from the Dana Farber Institute (http://compbio.dfci.harvard.edu/tgi/software/). Multiple sequence alignments were performed with the online tool PRRN using the default settings except for the gap open penalty, which was lowered to 7.0 to achieve better scoring alignments (http://www.genome.jp/tools/prrn/). To determine presence of rRNA in the subtracted library, a blastn search was performed against a eukaryotic rRNA database assembled from the European ribosomal RNA database (http://www.psb.ugent.be/rRNA/) with the recommended expect value (*e*-value) cutoff of 1e-65 [6]. Samples were similarly screened for mitochondrial RNA by searching a database of mitochondrial proteins curated by the National Center for Biotechnology and Information (NCBI) using the *e*-value cutoff of 1e-08 (ftp://ftp.ncbi.nlm.nih.gov/blast/db/FASTA/mito.aa.gz). All preprocessed EST contigs and singletons less than 200 base pairs in length were discarded. The term “unigene” will be used to refer to a contig or a singleton throughout this study.

### *EST annotation*

Annotations were assigned to ESTs in this study in three stages. Similarity search methods of extant protein databases generally produce more accurate annotations than *de novo* prediction methods [6]. We therefore annotated sequences using similarity searches of the Uniref100 database as the first stage. UniRef100 contains all the records in the UniProt knowledgebase and merges identical sequences and subfragments as a single entry, and thus increases speed and accuracy of homology searches [7]. Homology searches of Uniref100 were performed with the BLAST tool BLASTX [8], which translates the query to all 6 possible reading frames using an *e*-value cutoff of 1e-07. Sequences with no high-scoring pairs (HSPs) from the Uniref100 BLAST search were passed to the second annotation stage.

The second stage analysis was performed with two open-source software platforms: annot8r and prot4EST. Detecting the correct reading frame for each EST is essential for robust *de novo* function prediction. The prot4EST software package includes a pipeline to correct EST datasets for frame shifts [6]. Sequence data were run through the prot4EST pipeline prior to annotation. The annot8r package is a tool for assignment of Gene Ontology (GO), Enzyme Commission (EC) and Kyoto Encyclopaedia of Genes and Genomes (KEGG) annotations [9]. GO constitutes a controlled vocabulary to describe function and location of gene products (The Gene Ontology Consortium, 2000). EC is a hierarchical enzyme classification based on the type of reaction catalyzed [10]. KEGG annotates biochemical pathways for wholly sequenced genomes [11]. In a preprocessing step, annot8r automatically downloads relevant files and generates a reference database that stores UniRef100 entries with GO, EC and KEGG annotations. For each of the GO, EC and KEGG entries, annot8r extracts a specific sequence subset from the UniRef dataset based on matches to the reference database. These three subsets are then formatted for BLAST searches. To annotate sequence data, annot8r conducts BLASTX searches with the recommended minimum cutoff bit score of 55 against these three subsets. The BLAST results are then parsed and the corresponding annotations retrieved from the reference database.

In the final stage of our analysis we used InterProScan, which is a software package that integrates the common methodologies in the area of protein family, domain and motif detection. The InterProScan package identifies signatures from the InterPro member databases by applying disparate algorithms [12]; see Additional file 1 for a complete listing of available databases and applicable analysis tools. This additional annotation was attempted on each EST regardless of whether it appeared in the first or second stage BLAST searches, and therefore allowed the identification of more distant evolutionary relationships.

To quantify the effect of *B. bovis* on infected larvae, we focused on unigenes that were annotated with one of three terms from the GO ontology. The GO term “immune response” (GO:0006955) includes both innate and adaptive immune responses. The GO term “stress response” (GO:0006950) is defined as an exogenous change in state or activity of a cell or an organism as a result of a disturbance in its homeostasis. The GO term “defense response” (GO:0006952) is a specific (child) term of stress response defined as a triggered response to the presence of a foreign body or the occurrence of an injury; it also includes some responses of the innate immune system.

### Microarray design and analysis

Custom high-density single channel oligonucleotide arrays were constructed by NimbleGen Systems Inc. using 13,601 of the 13,642 members of BmiGI Version 2 and 14 perfect-match 50-mer probes per BmiGI target; these microarrays are described in detail by Saldivar *et al.*[4]. Probes with randomly generated sequences were designed into the arrays, but no mismatch probes were included. Each array chip includes two in-slide replicates, called spot replicates which have the same probe spotted on different locations on the chip, and are considered technical replicates as each of the probes for the 13,601 Gene Index members are spotted on different locations within the chip. The spot replicates increase precision and provide a basis for testing differences within treatment groups. Because of the status of *R. microplus* as an arthropod requiring adherence to strict United States Department of Agriculture (USDA) quarantine and handling restrictions and because of the need to sacrifice the calves at the end of each experiment, the ideal independent biological replicates were not available. Instead, we utilized pooled tick samples sourcing from an experiment with a single calf for each of the infected and uninfected feeding experiments. Our array experimental design consisted of four technical replicates, i.e. repeated measurements of the same *R. microplus* mRNA isolated from the feeding larval sample recovered and pooled from the single *B. bovis* infected or uninfected calf. In total, the design consisted of two chip replicates, each chip containing two spot replicates that are located on each chip. Samples were labeled before hybridization to the microarrays. After scanning the arrays, image analysis was conducted at NimbleGen Systems Inc. as described by Saldivar *et al.*[4]. Quality control measures and preprocessing were performed using the statistical computing language R and Bioconductor [13,14]. All microarray images and quality control measurements were within recommended limits, although one of the two replicate larval arrays was marginal. The quality of the arrays was assessed through standard quality control measures including: pseudo-images of the arrays (to detect spatial effects), scatter plots of the arrays versus a pseudo-median reference chip, and additional summary statistics including histogram, box plots of raw and normalized log intensities.

The intensity raw values were normalized using quintile normalization, gene calls generated using the Robust Multichip Average (RMA) algorithm [15,16], and raw intensity data log base 2 (log2) transformations [4]. The microarray datasets have been submitted to the GEO database (http://www.ncbi.nlm.nih.gov/geo/; GEO accession number GSE10816). The data files were loaded into Microarray Experiment Viewer (MeV Version 4.0, Dana-Farber Cancer Institute, Boston, MA, USA) and Significance Analysis of Microarrays (SAM) [17,18] selected statistically significant differentially expressed genes. A threshold value delta equal to 1.8 and a fold change ≥ 2.0 were used to separate significant from non-significantly differentially expressed ESTs. With the selected delta and fold change parameters, SAM estimated the proportion of false positives as < 0.00001.

### Verification by Real-Time PCR

Array results were selected for verification based on their level of differential expression and the amount of annotation available for their corresponding BmiGI sequence. The same total RNA samples used for the microarrays were also used for the real-time PCRs. Four micrograms of DNA-free total RNA for each sample was used according to manufacturer’s recommendations with the RETROscript Kit Reverse Transcription for RT-PCR (Ambion) to produce cDNA for each sample. Primers and TaqMan probes were designed using Beacon Designer 7.5 (PREMIER BioSoft International, Palo Alto, CA; Table 1) and synthesized by Sigma-Aldrich Inc. (Atlanta, GA) for each EST selected and for the *R. microplus* 18S rRNA gene, which was used as reference gene for normalization [4]. Validation and primer optimization experiments were run on each EST and reference gene to determine the efficiencies of the target ESTs and reference gene and optimal concentrations to be used in individual experiments. All real-time reactions were carried out in clear low 96 well plates (no. MLL9601, BioRad, Hercules, CA) and sealed with Microseal B film (BioRad) using 25μL total reaction volumes including primers, 250nM TaqMan probe, TaqMan Universal Master Mix No AmpErase UNG (Applied Biosystems Inc., Foster City, CA) and corresponding RETROscript cDNA. The final primer concentration for the 18S rRNA reference gene and the ESTs were 900nM for both the forward and reverse primers with the exception of 300nM for the TC9020 reverse primer. All primer and probe sequences are listed in Table 1. The BioRad CFX96 Real-Time System was used with a cycling protocol of 95^o^C for 10 minutes, 50 cycles of 95^o^C for 15 seconds, and 60^o^C for 1 minute plus plate read. The fluorescence emission data analysis was done using the baseline subtracted curve fit mode with CFX Manager Software version 1.0 (BioRad).

**Table 1:** Relative quantitative real-time PCR primers and probes

**EST**	**Primers**	**Taqman Probe**
TC293	FW 5’ AACATCTACAGCAAGTTTGACACC 3’	5’ FAM- AGGTGACCGCCTGGATACTCCGCA-TAMRA 3’
	RV 5’ CCCGTCGATGCAGGTTTTGG 3'	
BEAI101TR	FW 5’ CGGAAGAAACGAGAAATACGAGAC 3’	5’ FAM- TGCGTCGGCACACACTGGTACAGC-TAMRA 3’
	RV 5’ TACATGAGAACAGTAGCATATAGGG 3’	
TC12256	FW 5’ CTTCACATTCAACACGCCCTAC 3’	5’ FAM-AGCCACAGCAACGCCATCGCCG-TAMRA 3’
	RV 5’ AAAACCGCTACGGCAAATGC 3’	
TC14244	FW 5’ TTAAACATTCTTTCGCTCATCAGTC 3’	5’ FAM-CCGCACGACGCAAGCCGAAA-TAMRA 3’
	RV 5’ TACATGAGAACAGTAGCATATAGGG 3’	
18S	FW 5’ CCTGAGAAACGGCTACCACATC 3’	5’ FAM-AGGAAGGCAGCAGGCGCGC-TAMRA 3’
	RV 5’ GTGCCGGGAGTGGGTAATT 3’	

^a^ Based on BmiGI Version 2 designations (http://compbio.dfci.harvard.edu/tgi/cgi-bin/tgi/gimain.pl?gudb=b_microplus).

^b^ Forward (FW) & reverse (RV).

## Results and discussion

We studied differential gene expression in *R. microplus* associated with *B. bovis* infection to better understand the interplay between the larval life stage of the tick host and the invading apicomplexan pathogen as the infection process initially takes place. We infected tick larvae by allowing *B. bovis*-free larvae to feed upon a *B. bovis*-infected calf and for comparison, we repeated the protocol, allowing uninfected larvae to feed upon an uninfected calf. We used various analytical approaches to characterize infection-induced differential gene expression in these larvae.

### Microarray results

From the microarray experiments, 24 transcripts were expressed in larval tissues at a statistically significant (adjusted p-value <0.01) higher level in ticks feeding upon a *B. bovis*-infected calf contrasted to ticks feeding on an uninfected calf (Additional file 2). Six transcripts were expressed in larval tissues at a statistically significant (adjusted p-value <0.01) lower level in ticks feeding upon a *B. bovis*-infected calf contrasted to ticks feeding on an uninfected calf (Additional file 2). Tables 2 and 3 show the greatest up- and down-regulated transcripts, respectively, with fold-change and annotation data. As similarly reported by Saldivar *et al.*[4], a number of the significantly differentially expressed genes have no useful annotation. Three of the 20 transcripts in Table 2 and 4 of the transcripts in Table 3 lacked significant (*e-*value < 0.001) BLASTX hits.

**Table 2:** ** *R. microplus* microarray transcripts with highest statistically significant up-regulation associated with *B. bovis* infection**

**ID**^**a**^	**d**^**b**^	**FC**^**c**^	**BLASTX Annotation**			
			**Protein**	**Species**	**Accession Number**	**e-Value**
TC13643	7.3	5.0	Dihydrodipicolinate synthetase	*Bradyrhizobium sp.*	CCD90265.1	3e-07
TC11482	6.9	3.8	Cytochrome P450-like	*Phillyrea latifolia*	CAK18871.1	9e-19
TC5802	5.9	3.9	GGY domain protein	*Argas monolakensis*	ABI52689.1	3e-26
TC9132	5.6	2.7	Hypothetical protein LELG_02536	*Lodderomyces elongisporus*	XP001525979.1	1e-04
TC12256	5.6	7.6	Hemelipoglycoprotein precursor	*Dermacentor variabilis*	ABD83654.1	0.0
TC9012	5.3	3.7	Glutathione S-transferase	*Dermacentor variabilis*	ABB46494.1	3e-62
TC15029	4.9	7.8	Hemelipoglycoprotein precursor	*Dermacentor variabilis*	ABD83654.1	1e-106
TC9597	4.8	2.7	Esterase	*Ixodes scapularis*	XP002411693.1	2e-58
TC8343	4.8	3.2	Methyltransferase-like 7A	*Macaca mulatta*	NP001180640.1	2e-33
TC8407	4.8	3.8	Acyl-CoA synthetase	*Ixodes scapularis*	XP002433879.1	7e-62
TC13794	4.7	2.2	NSS^d^	*-*	-	-
BEADQ11TR	4.5	3.3	Non-LTR retrotransposon	*Bombyx mori*	BAD82945	2e-24
TC9454	4.5	5.0	Midgut cysteine proteinase 4	*Rhipicephalus appendiculatus*	AAO60047.1	0.0
TC5904	4.5	2.1	NSS	*-*	-	-
TC5872	4.5	2.9	GGY domain protein	*Argas monolakensis*	ABI52689.1	2e-12
TC10734	4.5	4.7	NSS	*-*	-	-
TC6802	4.4	2.1	Carboxypeptidase A2 precursor	*Ixodes scapularis*	XP002406597.1	2e-96
BEACP61TR	4.3	2.8	Microplusin	*Rhipicephalus microplus*	Q86LE5.1	2e-69
TC12142	4.2	3.2	Glycine-rich secreted protein	*Ixodes scapularis*	XP002411976.1	1e-10
TC12248	4.1	3.0	Mucin 68D	*Drosophila melanogaster*	NP648504.2	5e-07

^a^ The identification number from BmiGI Version 2 (http://compbio.dfci.harvard.edu/tgi/cgi-bin/tgi/gimain.pl?gudb=b_microplus).

^b^ d is the d statistic as performed by SAM.

^c^ FC is the fold change ratio.

^d^ NSS indicates no statistically significant similarity found in BLASTX search, based on *e*-value < 0.001.

**Table 3:** ** *R. microplus* microarray transcripts with highest statistically significant down-regulation associated with *B. bovis* **

**ID**^**a**^	**d**^**b**^	**FC**^**c**^	**BLASTX Annotation**			
			**Protein**	**Species**	**Accession Number**	**e-Value**
TC14933	−6.1	−2.6	Phosphatidylinositol 4-kinase	*Brassica napus*	AAQ24839.1	1e-06
BEAC172TR	−5.3	−4.6	NSS^d^	*-*	-	-
TC14244	−4.9	−3.4	NSS	-	-	-
TC12338	−4.7	−2.2	NSS	*-*	-	-
BEAC711TR	−4.5	−2.9	NSS	*-*	-	-
BEADW01TF	−4.4	−3.5	NSS	*-*	-	-

^a^ The identification number from BmiGI Version 2 (http://compbio.dfci.harvard.edu/tgi/cgi-bin/tgi/gimain.pl?gudb=b_microplus).

^b^ d is the d statistic as performed by SAM.

^c^ FC is the fold change ratio.

^d^ NSS indicates no statistically significant similarity found in BLASTX search, based on *e*-value < 0.001.

**Table 4:** GO annotation summary by domain

**Cellular component (C)**		
**GO ID**	**Description**	**Occurrences**
GO:0016020	Membrane	52
GO:0005623	Cell	15
GO:0005576	Extracellular region	15
GO:0005622	Intracellular region	132
**Molecular function (F)**		
**GO ID**	**Description**	**Occurrences**
GO:0003774	Motor activity	0
GO:0016874	Ligase activity	3
GO:0016829	Lyase activity	4
GO:0004871	Signal transducer activity	6
GO:0016491	Oxiodoreductase activity	24
GO:0016853	Isomerase activity	1
GO:0030234	Enzyme regulator activity	8
GO:0003824	Catalytic activity	36
GO:0005488	Binding	113
GO:0016740	Transferase activity	22
GO:0005198	Structural molecule activity	18
GO:0005215	Transporter activity	12
**Biological process (P)**		
**GO ID**	**Description**	**Occurrences**
GO:0006944	Cellular membrane fusion	3
GO:0050896	Response to stimulus	18
GO:0007610	Behavior	0
GO:0006810	Transport	26
GO:0030154	Cell differentiation	17
GO:0008152	Metabolic process	87
GO:0050789	Regulation of biological process	52
GO:0043062	Extracellular structure organization	0
GO:0007275	Multicellular organismal development	23
**GO:0009987**	**Cellular process**	**40**
**GO:0007154**	**Cell communication**	**3**
**GO:0008219**	**Cell death**	**3**
**GO:0006139**	**Nucleobase, nucleoside, nucleotide and nucleic acid metabolic process**	**49**

We attempted to identify genes whose transcripts played significant roles in the host-pathogen interactions between *B. bovis* and *R. microplus*. Our microarray approach identified infection-associated transcripts from a preexisting EST database, BmiGI Version 2.0. Interestingly, the second greatest up-regulated transcript in the larval microarrays, TC11482, had sequence similarity to cytochrome P-450, a family of genes whose products are often involved in a rapid response to external environmental stresses, including detoxification of xenobiotics (Table 2). The unigene TC9012 was also up-regulated in the microarrays and shared sequence similarity to glutathione S-transferase, another gene family often involved in detoxification or stress response. In fact, TC9012 has significant sequence similarity to the glutathione S-transferase DvGST2, which was reported as differentially up-regulated in *Dermacentor variabilis* in response to infection by *Rickettsia montanensis*[19]. DvGST2 was also reported as up-regulated upon blood-feeding in the same tick species [20]. The glutathione S-transferase induction, and those of the P-450 transcript discussed above, may be in response to toxic heme by-products of bloodfeeding, rather than to *B. bovis* infection. However, the uninfected sample controls were at similar developmental stages and actively ingesting blood. Indeed, the heme-induced responses should have been cancelled out in the comparison between control and infected samples. The *B. bovis* infection is likely imposing unique stresses within the ticks that are responsible for these detoxification responses being induced in a tissue-specific manner.

A small number of differentially expressed transcripts were observed when contrasted with other microarray studies using the same BmiGI Version 2-derived arrays. We found 30 statistically significant differentially expressed transcripts in our larval microarray experiments. Mercado-Curiel and colleagues measured the temporal response of gene expression in adult male *R. microplus* in response to *Anaplasma marginale* infection [21]. When they compared infected ticks with uninfected controls using microarray assays, they determined that 888 genes were differentially expressed in midgut tissue 2 days post-infection and 146 genes were differentially expressed in the salivary glands 9 days post-infection. In contrast, Ribeiro and colleagues found only 10 differentially expressed genes in the salivary glands of *Ixodes scapularis* nymphs in response to *Borrelia burgdorferi* infection [22]. Rodriguez-Valle *et al.* found over 300 differentially expressed BmiGI transcripts in their study of *R. microplus* feeding upon *Bos indicus* and *Bos taurus* cattle [23]. Feeding upon tick resistant cattle as opposed to tick susceptible cattle evidently creates greater perturbations compared with feeding upon *B. bovis*-infected blood as opposed to uninfected cattle blood. Saldivar *et al.* discovered 76, 32, 80, and 83 differentially expressed BmiGI Version 2 transcripts in their microarray analysis of gene expression changes in response to larval exposure to the acaricides: coumaphos, permethrin, ivermectin and amitraz, respectively [4].

### Subtracted library results and discussion

ESTs from the *B. bovis*-infected larval subtracted libraries were assembled and annotated. Additional file 1 contains data pertaining to annotated unigenes. Of the 791 total transcripts sequenced, 30 were classified as coming from *B. bovis* and removed from analysis. Three additional sequences, one from a mitochondrial gene, one rRNA sequence, and one putative transcript from *Bos Taurus* were also removed. Of the remaining 758 sequences, 193 had little sequence similarity to BmiGI Version 2 transcripts and therefore appear to be novel. The Uniref100 database searches revealed 381 sequences with similarity to known proteins.

### *GO annotations*

The annot8r application provides a synopsis of the GO annotation process by categorizing the unigenes into 3 domains consisting of 29 high-level GO terms (Table 4). In the cellular component domain (C), most of the differentially expressed transcripts were deemed intracellular. The predominant annotation in the molecular function domain (F) was protein binding followed by catalytic activity and oxidoreductase activity. Metabolic processes were the largest component of the biological process domain (P).

We further utilized the GO annotations to find unigenes with possible roles in the infection process [24,25]. Gene products identified via BLASTX searches with GO annotations of defense response (GO:000692) or stress response (GO:006950) are listed in Tables 5 and 6, respectively. Each unique annotation comprises the study sequence identifier, the source (database) of the annotation, a description of the annotation, the genus and species of the HSP, the NCBI accession number of the protein, and the corresponding e-value for the HSP.

**Table 5:** Unigenes from subtracted library with BLAST database GO annotation of defense response (GO:0006952)

**Unigene #**^**a**^	**Database**	**BLASTX Annotation**			
		**Protein Annotation**	**Species**	**Accession Number**	** *e* -Value**
45	Uniref100	Salivary lipocalin	*Amblyomma variegatum*	DAA34698.1	2e-07
92	KEGG	Toll-like receptor signaling pathway	*Ixodes scapularis*	XP002404081.1	5e-176
113	Uniref100	Interferon gamma-inducible protein	*Amblyomma americanum*	AAK82985.1	2e-92
402	Uniref100	Glycoprotein 3-alpha-l-fucosyltransferase A	*Ixodes scapularis*	XP002413615.1	3e-70
468	Uniref100	Putative defense protein precursor	*Bombyx mori*	NP001091819.1	2e-20
673	Uniref100	FYN binding protein	*Ixodes scapularis*	XP002412475.1	1e-22

^a^ Unigene identification number as listed in Additional file 1.

**Table 6:** Unigenes from subtracted library with BLAST database GO annotation of stress response (GO:0006950)

		**BLASTX Annotation**			
**Unigene #**^**a**^	**Database**	**Protein Annotation**	**Species**	**Accession Number**	** *e* -Value**
40	Uniref100	Glutathione Peroxidase	*Ixodes scapularis*	XP002399259.1	1e-41
79	Uniref100	Valacyclovir hydrolase	*Ixodes scapularis*	XP002404467.1	2e-56
89	GO	Hydrogen peroxide catabolism	*Homo sapiens*	A1KZ92	1e-11
187	Enzyme class	Peroxidase	*Caenorhabditis elegans*	Q1EN18	4e-10
215	Uniref100	Heat shock protein	*Locusta migratoria*	AAO21473.1	4e-10
244	Uniref100	Cytochrome P450	*Ixodes scapularis*	B7PSW2	1e-81
416	Uniref100	Thioredoxin domain-containing protein	*Ixodes scapularis*	XP002436084.1	9e-28
489	Uniref100	Cytochrome P450	*Ixodes scapularis*	XP002414034.1	2e-44
637	Uniref100	Bleomycin hydrolase	*Ixodes scapularis*	XP002399259.1	1e-78

^a^ Unigene identification number as listed in Additional file 1.

Annotations for 6 unigenes from the assembled subtracted library sequencing were related to defense response by similarity to proteins in the Uniprot database annotated by GO (Table 5). Among the 6 ESTs, 2 were homologous to proteins in *Ixodus scapularis*. Unigene 402 showed significant similarity to 3-alpha-1-fucosyltransferase, which has been demonstrated to increase microbial pathogenesis in *I. scapularis*[26]. Unigene 468 was identified as a putative defense protein precursor by similarity to a protein from *Bombyx mori.* Two defense related hydrolases were also identified. Valacyclovir hydrolase (unigene 79) is designated as “response to toxin” by the GO ontology (GO:0009636). In addition, Bleomycin hydrolase (unigene 637) inactivates bleomycin B2 (a cytotoxic glycometallopeptide: GO:0009636). Unigene 113 displayed similarity to an Interferon gamma-inducible protein that in humans has a role in antigen processing and epitope presentation [27], but in ticks may upregulate cathepsins that activate serine proteases as an immune response. A putative salivary lipocalin (unigene 45) suggests a role in the tick’s immune response since lipocalins are involved in many inflammation and detoxification processes in mammals [28]. Unigene 673 is a putative FYN binding protein; the GO Ontology annotates this protein with the term “defense response”. As determined by BLAST search similarity to the KEGG database, Unigene 92 exhibited strong homology (*e*-value = 5e-176) to an *I. scapularis* protein within the Toll-like receptor-signaling pathway—an important component of the host innate immune system.

Annotations for 9 unigenes from the assembled subtracted library sequencing subsumed the GO term “response to stress” (GO:0006950) by similarity to proteins in the Uniprot database (Table 6). Moreover, 6 unigenes were homologous to proteins in *I. scapularis*. Unigenes 244 and 489 showed significant similarity to two *I. scapularis* cytochrome P450 proteins, which are potent detoxifiers of xenobiotics. Other observed detoxification proteins include: thioredoxin (unigene 416) and glutathione peroxidase (unigene 40), which facilitate the reduction of proteins and lipids, respectively. Two putative antioxidant peroxidase-related proteins (unigenes 89 and 187) were also identified. Two putative stress response-related hydrolases were detected in the subtraction library. Valacyclovir hydrolase (unigene 79) is annotated with the biological process “response to toxin” (GO:0009636). In addition, Bleomycin hydrolase (unigene 637) inactivates bleomycin B2 (a cytotoxic glycometallopeptide). Unigene 215 is homologous to a putative heat shock protein discovered in *Locusta migratoria*. Heat shock protein expression levels are generally upregulated when the organism is stressed.

The InterProScan algorithms yielded an additional 190 unique sequence annotations, which brought the total number of annotated ESTs to 571 out of 758 (75%). No unigenes were annotated with the term “immune response” (GO:006950) by homology searches. However, Table 7 lists unigenes associated with immune function identified by InterProScan. Each unique annotation comprises the study sequence identifier, the protein, source (method) of the annotation, and the InterPro identifier assigned to this protein. InterProScan annotated 11 unigenes with immune or defense response-related function including an additional lipocalin (unigene 443) that was not identified in the BLAST searches. Six of these unigenes were classified as immunoglobulin or immunoglobulin-like proteins. Since the tick has no adaptive immune system, we speculate that these proteins are either false positives or may serve a similar role to proteins that have homologs in vertebrates. For example, the human Down Syndrome cell adhesion molecule (DSCAM) has an immunoglobulin-like domain with several known homologs in arthropods that may have thousands of splice variants and help drive the clearance of pathogens [29,30].

**Table 7:** Unigenes from subtracted library with InterProScan annotation of immune response

	**InterProScan Annotation**		
**Unigene #**^**a**^	**Protein Annotation**	**Methods**	**Interpro ID**
298	Serine Protease Inhibitor	HMMPfam, superfamily	IPR002919
371	Serine Protease Inhibitor	HMMPfam, superfamily	IPR002919
520	Serine Protease Inhibitor	HMMPfam, superfamily	IPR002919
89	Immunoglobulin-like	HMMPfam, HMMSmart, Gene3D, ProfileScan	IPR007110
187	Immunoglobulin-like	HMMPfam, HMMSmart, Gene3D, ProfileScan	IPR002919
216	Immunoglobulin	Superfamily	
288	Immunoglobulin-like fold	HMMPfam, HMMSmart, Gene3D, ProfileScan	IPR013783
520	Immunoglobulin-like	HMMPfam, HMMSmart, Gene3D, ProfileScan	IPR002919
601	Immunoglobulin-like	HMMPfam, HMMSmart, ProfileScan, Superfamily	IPR007110
242	Lectin/glucanase	Gene3D, Superfamily	IPR008985
443	Lipocalin	Superfamily	IPR011038

^a^ Unigene identification number as listed in Additional file 1.

**Figure 1:**
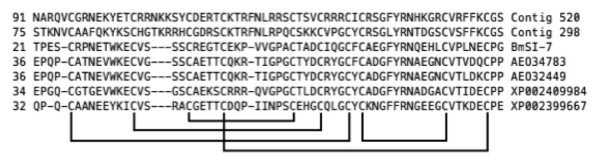
**Multiple sequence alignment of two putative serpins identified in the subtracted larval library with a known serpin from *R. microplus* (BmSI-7), two putative serpins from *Amblyomma maculatum* (AEO34783 and AEO32449) and two putative serpins from *Ixodes scapularis* (XP002409984 and XP002399667).** The five disulfide bridges formed by 5 pairs of cysteine residues are indicated by the black lines. The numbers to the left are the position of the first amino acid (first column) within each polypeptide.

The remaining three unigenes were characterized as serine protease inhibitors (serpins). Recent interest in tick serpins stems from their significant antimicrobial and antifungal activity [31-33]. Serpins are cysteine-rich antimicrobial peptides and are components of the immune system of many invertebrates. Serpins have been used as target antigens for recombinant tick vaccines [34]. Unigenes 298 and 520 had sequence similarity (HSPs) to proteins that belong to the Trypsin inhibitor like cysteine rich domain (TIL) family. Huang *et al.* characterized a protein, C/E1 from *Ascaris suum*, with a TIL domain about 60 residues long containing five disulfide bonds - two of which are located on either side of the reactive site. Serpins with this domain were subsequently isolated and verified in *R. microplus*[35]. Figure 1 shows a multiple sequence alignment of unigenes 298 and 520 with a validated serpin from *R. microplus* (BmSI-7), two putative serpins from *Amblyomma maculatum* (AEO34783 and AEO32449) and two putative serpins from *Ixodes scapularis* (XP002409984 and XP002399667). The figure demonstrates that the portions of each protein containing the TIL domain, including the 5 cysteine residue pairs that form the disulfide bridges, are well aligned. Note that these protein domains were *predicted* by InterProScan and not found in the BLASTX search of the Uniprot100 database, thus underscoring the utility of *de novo* domain prediction algorithms.

Other noteworthy genes include three novel unigenes involved in signal transduction pathways. Unigene 192 was predicted to be low-density lipoprotein receptor-related by InterProscan. The GO annotations included the term extracellular region (GO:0005576), hemoglobin import (GO:0020028) and lipoprotein transport (GO:0042954). This protein likely performs some binding function involved in the uptake of lipoproteins related to feeding on blood. Unigene 331 is a predicted membrane protein by InterProScan that is involved in ion transport. The BLAST annotation characterizes the protein as a voltage sensitive phosphatase and therefore may be involved in neural signal transduction. Unigene 412 is a putative porcupine-like protein that is integral to the Wnt signaling pathway. InterProScan predicted transmembrane regions in this protein including a signal peptide, which is further evidence of its relationship to membrane signal transduction.

## *PCR results*

To verify the microarray results and the subtraction library results, we selected 2 transcripts from each result data set with significant differential expression of varying fold-changes and performed qRT-PCR to compare transcript levels in the *B. bovis* infected larval tissue to the uninfected larval controls. Table 8 shows that the directional expression changes for the selected transcripts were qualitatively similar in both the microarrays and qRT-PCRs. Table 9 likewise indicates that directional expression changes for the selected transcripts were qualitatively similar in both the subtracted library and qRT-PCRs.

**Table 8:** RT-PCR verification of selected microarray results

**EST**	**Microarray**		**Relative Quantitative PCR**	
	**Uninfected**	**Infected**	**Uninfected**	**Infected**
TC12256	1.0	7.6	1.0	8.4
TC14244	3.4	1.0	1.4	1.0

^a^ Normalized data to set lower value to 1 for comparison purposes.

**Table 9:** RT-PCR verification of selected subtraction library results

**Unigene #**^**b**^	**Relative Quantitative PCR**	
	**Uninfected**	**Infected**
298	1.0	1.9
520	2.1	1.0

^a^ Normalized data to set lower value to 1 for comparison purposes.

^b^ Unigene identification number as listed in Additional file 1.

## Conclusion

In summary, our experimental approaches yielded specific differential gene expression associated with the infection of *R. microplus* larvae by *B. bovis*. However, the number of statistically significant differentially expressed transcripts was lower than we had anticipated. Microarray experiments determined that 24 transcripts were expressed in larval tissues at a statistically significant higher level in ticks feeding upon a *B. bovis*-infected calf contrasted to ticks feeding on an uninfected calf. Six transcripts were expressed in larval tissues at a statistically significant lower level in ticks feeding upon a *B. bovis*-infected calf contrasted to ticks feeding on an uninfected calf. Although the BmiGI Version 2 gene index was a useful database to help assign putative function to specific transcripts, a significant percentage of the differentially expressed transcripts did not have annotation that was useful for assignment of function. This emphasizes the need for further investigation of the genome of the cattle tick to develop a resource containing the full transcriptome. Our database of differentially expressed genes responding to *B. bovis* infection will be used to guide further investigations of the *B. bovis*-*R. microplus* complex.

The diversity of our approaches provides a good database of transcripts that express differential regulation in response to *B. bovis* infection. The ESTs from the subtracted libraries add to the list of genes involved in the tick infection process and we focused on transcripts related to the ticks’ stress response and innate immune response. The functions of these transcripts might provide insight into the infection and transmission processes of *B. bovis* as it interacts with its host tick, *R. microplus*.

The subtraction library yielded 28 unique transcripts related to immune, defense, and stress responses, which implies an up-regulation of expression levels of the corresponding genes in response to *B. bovis* infection. It is also lends insight into the defense mechanisms at the disposal of *R. microplus* while still in the larval stage of development.

## Competing interests

The authors declare that there are no competing interests.

## Authors’ contributions

FDG conceived the study, participated in the design, data collection, and analysis of the study and drafted the manuscript. AMH performed bioinformatic analysis of the ESTs and drafted the manuscript. KGB participated in the data collection, data analysis, and designed the RT-PCR verification study. LS and CG participated in analysis of the microarray data; GAS participated in the overall study design, infection of cattle, timing of tick infections and collection of tick materials. VN, SED and AD participated in study design and coordinated the sequencing phases. KAB participated in study design and microarray experimental design. All authors read and approved the final manuscript.

## Supplementary Material

Additional file 1 **ESTs from the *Babesia bovis* -infected larvae subtracted library.** This Excel file contains EST sequences from the subtracted library synthesized from the *Babesia bovis*-infected larvae (using uninfected larvae for the subtraction) and BLASTX annotation information including: GO, EC, KEGG, and InterProScan generated annotations. Click here for file

Additional file 2 **BmiGI entries significantly up- or down-regulated in microarray experiments.** Analysis of microarray data resulted in identification of specific entries from the *Rhipicephalus microplus* gene index, BmiGI Version 2, that are statistically up- or down-regulated in response to *Babesia bovis* infection. This Excel file contains BmiGI ID number, hit descriptions, annotation, GO Terms, *e*-values, reading frames, and related information. Click here for file
